# Risk Factors for Poor Adherence to Betaferon^®^ Treatment in Patients with Relapsing-Remitting Multiple Sclerosis or Clinically Isolated Syndrome

**DOI:** 10.1371/journal.pone.0157950

**Published:** 2016-10-03

**Authors:** Łukasz Jernas, Jacek Wencel, Andrzej Wiak, Marek Bieniek, Halina Bartosik-Psujek

**Affiliations:** 1 Department of Neurology, Poznań University of Medical Sciences, Poznań, Poland; 2 Department of Neurolofy, "L. Bierkowski" Ministry of Internal Affairs and Administration Health Care Centre in Poznan, Poznań, Poland; 3 Department of Neurology, 4th Military Clinical Hospital in Wrocław, Wrocław, Poland; 4 Bayer Sp. z o.o, Pharmaceuticals, Medical Department, Warsaw, Poland; 5 Faculty of Medicine. University of Rzeszow, Rzeszów, Poland; University of Oxford, UNITED KINGDOM

## Abstract

**Introduction:**

Adherence to treatment, including early treatment discontinuation, in patients with multiple sclerosis or clinically isolated syndrome can be affected by: treatment tolerability, route of drug administration, patient age, disease duration, comorbidities, medical care, and support from their caregivers.

**Aim:**

This study aimed to identify the risk factors for poor adherence to Betaferon^®^ treatment, including early discontinuation and omitting doses.

**Materials and Methods:**

852 adult patients treated with Betaferon participated in this 24-month study. All patients were interviewed using the Risk of Drop-out Questionnaire, the Center for Epidemiologic Studies Depression Scale and the Kurtzke Expanded Disability Status Scale.

**Results:**

Patients who stopped therapy were younger (p = 0.003) had a higher mean EDSS score (p = 0.022), higher mean number of relapses (p = 0.017), and reported more often fear of injection (p = 0.027) and adverse events (p = 0.007) than those who did not stop treatment. Comparing patients who stopped therapy in the first and the second year, patients who stopped therapy in the first year of treatment more frequently reported flu-like symptoms and fever, and those who stopped therapy in the second year reported—ineffectiveness of treatment and disease progression. Multivariable logistic regression models confirmed that young age, short disease duration, advanced and progressing disease, and poor Betaferon tolerability were related to premature treatment discontinuation. The risk of omitting a dose during therapy was increased in patients who were working or studying, who had more advanced disease or more adverse events, and in patients who received less support from their caregivers.

**Conclusions:**

Several reasons may lead to problems with adherence to Betaferon treatment. Patients at higher risk of discontinuing treatment need to be identified early to make caregivers’ support available to them.

## Introduction

Multiple sclerosis (MS) is an autoimmune inflammatory demyelinating disease of the central nervous system. The clinical presentation and course of the disease are variable but in most cases it leads to advanced disability. Several disease types can be recognised: relapsing–remitting MS (RRMS), primary–progressive, secondary–progressive, progressive–relapsing MS and clinically isolated syndrome (CIS) [1,2].

The treatment of MS varies depending on individual disease characteristics. Patients with RRMS are treated with immunomodulatory agents including: interferon beta preparations [3,4], glatiramer acetate [5], dimethyl fumarate [6,7], natalizumab [8], fingolimod [9,10], and teriflunomide [11,12,13]. Many clinical trials show that early treatment with interferon (IFN) [e.g. IFNβ-1b (Betaferon^®^)] reduces the relapse number, and delays conversion to clinically definite MS [14,15,16]. IFN treatment also reduces relapse severity, hospitalisations, disease activity as assessed by magnetic resonance imaging (MRI), and increases the time to first relapse.

Drugs used to treat MS have different routes of administration (oral or injection), different frequency of use, and varying side effects. As a result, some patients may omit single doses or even totally discontinue therapy.

Non-adherence to the injection regimen is one of the problems with patient care, especially with interferon therapy. Studies performed to date have shown that 13–30% of patients discontinued therapy [17]. As well as the route of administration and side effects mentioned before, other factors influencing discontinuation of treatment include age, time since disease onset, and comorbidities. In addition, medical care support plays an important role in adherence to therapy. In the START study, Dhib-Jalbut et al. showed that if patients received nursing support, combined with dose titration and use of analgesics, the majority would adhere to therapy [18]. Therefore, appropriate nursing care is important in a patient’s adherence to the treatment regimen and the regularity of drug administration. A properly trained nurse, dedicated to caring for patients with MS, carefully supervising therapy and working closely with the responsible physician, can significantly improve the efficacy of treatment.

### Aim

The objective of this study was to identify the risk factors in patients with MS or CIS for early discontinuation of treatment with Betaferon and a lack of persistence (irregular administration of the drug).

The following risk factors were considered: adverse events, technical aspects of injection, support provided to the patient (at the study centre and at home), stage of the disease, importance of treatment with Betaferon as perceived by the patient, and symptoms of depression.

## Materials and Methods

852 adult patients diagnosed with RRMS or CIS, who decided to start immunomodulatory treatment with Betaferon^®^ (Bayer Pharma AG), participated in the study. Patients were included if they had started treatment with Betaferon no earlier than 60 days before enrolment to the study. Study was approved by Bioethics Committee of Medical University of Lublin, and informed consent was obtained from participants in writing.

All patients were evaluated during an interview about risk factors associated with discontinuation of treatment with Betaferon and a lack of persistence. The Risk of Drop-out Questionnaire was completed once per month by the nurse supervising drug administration. Depression was assessed as a separate risk factor using the Center for Epidemiologic Studies Depression Scale (CESD-R). Level of disability was measured using the Kurtzke Expanded Disability Status Scale (EDSS).

The study was carried out for 24 months and included a baseline visit (Visit 1) and four follow-up visits (at 6, 12, 18 and 24 months after Visit 1). During Visit 1, the following data were collected: patient demographics, employment status, care giver details, family history of MS, and the medical history relating to MS (including onset of first symptoms, date of MS diagnosis, location of first symptoms, form of MS, number of relapses within the previous 2 years, and an MRI of the brain was made. In addition, the current level of disability was assessed using the EDSS and use of disease-modifying medications noted.

At the next three visits, the following were noted: the number of new relapses since the previous visit, the level of disability according to the EDSS, changes in immunomodulatory treatment, adverse events, and depression was assessed using the CESD-R; an MRI of the brain was also made. At the last visit (Visit 5), the date Betaferon treatment ended was recorded, and further information provided for continuing treatment after the 24 months.

The nurse was responsible for monthly control of the quantity of administered drug and completing the Risk of Drop-out Questionnaire every 3 months.

### Applied questionnaires

#### Risk of Drop-out Questionnaire(RODQ)

The RODQ was originally developed by Bayer Schering Pharma to be used by nurses to identify when patients on long-term Betaferon^®^ treatment might run into difficulties with adherence and to estimate the risk of treatment termination. The RODQ comprises 12 items on 5 subscale items rated on a 3-point scale. The subscales refer to the current situation and satisfaction of the patient with the Betaferon treatment: 1. Side effects, 2. Injections and Handling, 3. Trust and Support, 4. Disease-related factors, 5. Importance of treatment. The disease-related factors (subscale 4) include rate of progression, recent relapse activity as reported by the patient as well as fatigue and depression as factors known to be relevant to treatment adherence. For the assessment of fatigue and screening for depression within the scope of the RODQ the Fatigue Visual Numeric Scale and the Two Question Test were applied. The Fatigue Visual Numeric Scale is a modified version of the fatigue visual analog scale with the score being a number between 0 (no fatigue) and 10 (worst imaginable fatigue) to be circled by the patient when requested by the nurse, with the higher score indicating more fatigue. The Two Question Test is a case-finding instrument for detecting depression. A “yes” answer to either of the following questions is considered a positive test: 1) During the past month, have you often been bothered by feeling down, depressed, or hopeless? or 2) During the past month, have you often been bothered by little interest or pleasure in doing things?

For the remaining subscales of the RODQ the nurse asked the patient open-ended questions and, according to the patient’s response, decided to tick one of the three possible answers per question. The number of and reason for a lack of persistence, and the need for using non-steroidal anti-inflammatory drugs, were also analysed.

#### Center for Epidemiologic Studies Depression Scale (CESD-R)

The 20-item CESD-R scale measures symptoms of depression in nine different dimensions as defined by the American Psychiatric Association Diagnostic and Statistical Manual, fourth edition. The dimensions are as follows: sadness (dysphoria), loss of interest (anhedonia), appetite, sleep, thinking/concentration, guilt (worthlessness), tiredness (fatigue), movement (agitation), and suicidal ideation. The Total CESD-R Score was calculated as a sum of responses to all 20 questions [19, 20].

### Statistical analysis

Statistical analysis was performed using R Statistical Software. All statistical tests were performed at the 0.05 significance level.

For variables from the RODQ, integer encoding was used (numbers from 1 to 3 were assigned to successive categories of variables), and a mean of the patients’ responses to each item was calculated.

To compare patients groups, the Mann–Whitney U test (for continuous variables) and Pearson's chi-squared test (for categorical variables) were used.

Multivariable logistic regression models were used in the analyses of the effect of the risk factors on premature discontinuation of treatment and frequency of omitted injections. A target binary variable was applied for logistic models determining risk factors associated with premature discontinuation of therapy: 1 for ‘premature discontinuation of treatment’ and 0 for ‘no premature discontinuation of treatment’. Logistic models used for identification of variables that affect the frequency of omitted injections were based on the target binary variable: 1 for ‘at least one dose omitted’ and 0 for ‘no dose omitted’.

#### Multivariable logistic regression models analysing the risk factors on premature discontinuation of treatment and the risk of lack of persistence during therapy

Two models were developed based on the results of the surveys: a model for defining the risk of premature discontinuation of treatment with Betaferon (Model 1) and a model defining the risk of lack of persistence (Model 2).

The following variables were considered for the models: age, sex, activity status (1 for working or studying patients and 0 for others), care status (1 if there was a person taking care of the patient and 0 for others), time since first symptoms occurred, time since diagnosis, number of relapses within the previous 2 years, EDSS (assessed at the first visit), EDSS difference (between the last and first visit), CESD-R (assessed at the first visit), CESD-R difference (between the last and first visit), mean number of new relapses, mean number of visits with adverse events reported, as well as variables from the RODQ.

## Results

### Baseline characteristics of the patients

Of the 852 patients in the study, data from 649 were analysed: 472 women and 177 men. 203 patients were excluded from the analysis because of the following reasons: lack of consent documentation, a diagnosis other than that specified in the inclusion criteria, start of Betaferon treatment earlier than 60 days before enrolment into the study, previous treatment with an immunomodulatory drug other than Betaferon, start of treatment with Betaferon 3 months or later after Visit 1, or the beginning of therapy was unconfirmed.

429 (66.1%) patients were working, 72 (11.1%) were students, 85 (13.1%) did not work because of the disease, and 54 (8.3%) did not work but this was not disease related. No information was available for 9 patients (1.4%). 499 patients (76.9%) did not require the help of others, while the remaining patients (23.1%) needed care from family members. 38 patients (5.9%) had a family history of MS. In three cases, brothers also had the disease; in four cases, sisters had MS; in five cases, the mothers had MS; in eight cases, the fathers had the disease. 637 patients (98.2%) had RRMS, and the rest had CIS.

### Relapses and EDSS score

The mean age of patients at baseline was 33.0 (SD 8.36) years. The mean time since the onset of symptoms was 3.7 (SD 4.08) years, and the mean duration of the disease calculated since the diagnosis was 2.1 (SD 3.45) years. The mean time between the first symptoms and the diagnosis was 1.6 (SD 2.49) years, the mean number of relapses during the 2 years since study enrolment was 2.1 (SD 1.00), and the mean EDSS score was 1.5 (SD 1.06). During Visit 2, the mean number of relapses was 0.1 (SD 0.35) and the mean EDSS score was 1.5 (SD 1.10). At Visit 3, the mean number of relapses was 0.1 (SD 0.44) and the mean EDSS score was 1.5 (SD 1.15). At Visit 4, the mean number of relapses was 0.1 (SD 0.36) and the mean EDSS score was 1.5 (SD 1.14). At Visit 5, the mean number of relapses was 0.1 (SD 0.31) and the mean EDSS score was 1.4 (SD 1.14).

### MRI

An MRI of the head was made for almost all participants: 98.5% during Visit 1, 10.8% during Visit 2, 41.8% during Visit 3, 17.1% during Visit 4, and 58.6% during Visit 5. The Gadolinium (Gd+)-enhanced lesions were observed in 10.3–31.4% of patients.

### Patient follow-up—treatment discontinuation and a lack of persistence

#### A. Patient follow-up—treatment discontinuation

**Fig 1:**
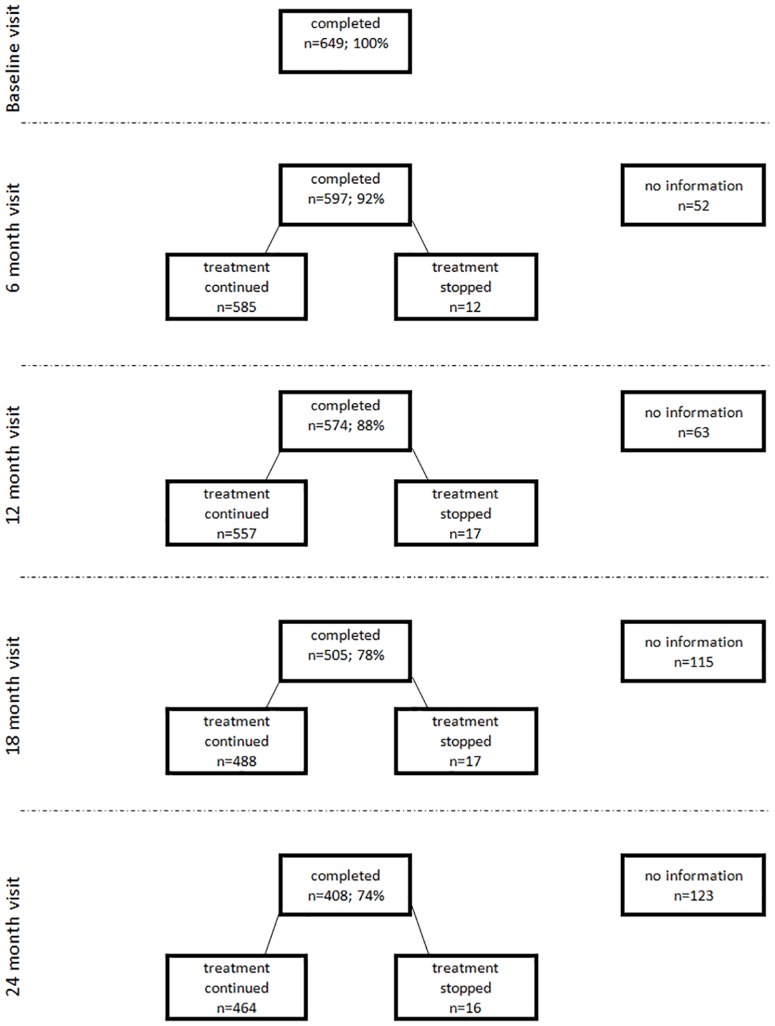
Patient flow chart during 24 months of follow-up.

The number of study participants decreased at each subsequent visit because of several reasons (Fig 1).

There were a few differences between patients who stopped the therapy compared with those who did not (Table 1). Those who stopped were younger than those who did not stop treatment (30.8 vs 33.7 years; p = 0.003). Patients who stopped therapy also displayed a higher mean EDSS score at the first visit (1.77 vs 1.40; p = 0.022), indicating worse physical ability, and showed a progression in disability compared with those who did not stop therapy. The mean EDSS difference between the first and last visits was 0.58 for those who stopped therapy early and -0.03 for those who did not stop therapy (p<0.001). Patients who stopped therapy experienced a higher mean number of relapses than those who continued (0.32 vs 0.11; p = 0.017). These patients also reported fear of injection (1.21 vs 1.12; p = 0.027) and adverse events more often (1.41 vs 1.24; p = 0.007) (Table 1).

**Table 1:** Differences between MS patients who stopped Betaferon therapy and those who did not.

		Patients who did not stop therapy		Patients who stopped therapy		Total
	Number of patients	464		62		526^1)^
		Mean	SD	Mean	SD	p-value
	**Age (years)**	33.7	8.17	30.8	8.13	0.003*
	**Time since first symptoms (years)**	3.69	4.187	2.72	3.215	0.066
	**Time since diagnosis (years)**	2.09	3.562	1.49	2.196	0.691
	**Time since symptoms to diagnosis (years)**	1.63	2.520	1.23	2.142	0.554
	**Number of relapses during 2 years preceding starting the study**	2.04	1.010	2.16	0.968	0.365
	**EDSS first visit**^2)^	1.40	1.011	1.77	1.210	0.022*
	**EDSS last visit**^3)^	1.38	1.067	2.35	1.747	<0.001
	**EDSS difference**	-0.03	0.605	0.58	1.153	0.000*
	**CESD first visit**^2)^	16.9	6.89	17.4	5.61	0.297
	**CESD last visit**^3)^	15.8	6.73	16.9	6.23	0.140
	**CESD difference**	-1.32	7.680	-0.72	5.319	0.993
	**Mean number of relapses since last visit**	0.11	0.192	0.32	0.560	0.017*
	**Mean number of visits with adverse events reported**	0.03	0.094	0.22	0.352	<0.001
**Patient-reported problems**^4)^	**Injection site reactions**	1.26	0.314	1.36	0.518	0.947
	**Other adverse events: flu-like symptoms, fever**	1.24	0.281	1.41	0.458	0.007*
	**Fear of injection**	1.12	0.221	1.21	0.302	0.027*
	**Practical application of injections**	1.04	0.120	1.11	0.279	0.675
	**Help from medical centre and neurologists**	1.03	0.118	1.02	0.102	0.279
	**Help from caregivers**	1.06	0.187	1.06	0.204	0.459
	**Significance of treatment**	1.08	0.187	1.12	0.220	0.151

^1)^ N = 123 patients were lost to follow-up

^2)^ First visit, Visit 1: start of therapy

^3)^ Last visit, Visit 5: 2 years after start therapy

^4)^ Mean was calculated based on all answers were coded as follows: 1. No problem/good support/very important; 2. Some problems/support might be better/important as part of treatment; 3. Serious problems/no support/not too important

* Statistically significant: p<0.05

Patients who stopped therapy in the first year differed from those who stopped treatment in the second year (Table 2). Patients who stopped therapy in the first year of treatment more often reported adverse events, such as flu-like symptoms and fever. However, the mean number of visits where adverse events were reported did not differ between patients who stopped Betaferon therapy and those who did not. Patients who stopped therapy in the second year decided to do so mainly because of ineffectiveness of treatment and deterioration of status as shown by reported mean EDSS score at the last visit (Table 2).

**Table 2:** Differences between patients who stopped therapy in the first and second year of therapy.

		Patients who stopped therapy in the first year		Patients who stopped therapy in the second year		Total
	Number of patients	29		33		62
		Mean	SD	Mean	SD	p-value
	**Age (years)**	30.34	8.739	31.21	7.672	0.489
	**Time since first symptoms (years)**	1.72	1.791	3.63	3.916	0.016*
	**Time since diagnosis (years)**	0.83	1.466	2.09	2.570	0.014*
	**Time since symptoms to diagnosis (years)**	0.9	1.145	1.55	2.755	0.256
	**Number of relapses during 2 years preceding starting the study**	2.04	0.978	2.26	0.965	0.409
	**EDSS first visit**^1)^	1.5	1.217	2.02	1.169	0.044*
	**EDSS last visit**^2)^	1.91	1.669	2.74	1.746	0.044*
	**EDSS difference**	0.41	1.044	0.73	1.238	0.083
	**CESD-R first visit**^1)^	18.1	5.856	16.89	5.473	0.452
	**CESD-R last visit**^2)^	16.75	6.357	17.04	6.260	0.821
	**CESD-R difference**	-1.55	3.502	-0.08	6.374	0.717
	**Mean number of relapses since last visit**	0.24	0.649	0.4	0.465	0.012*
	**Mean number of visits with adverse events reported**	0.34	0.445	0.1	0.186	0.058
**Patient-reported problems**	**Injection site reactions**	1.4	0.608	1.32	0.435	0.83
	**Other adverse events: flu-like symptoms, fever**	1.57	0.561	1.28	0.299	0.043*
	**Fear of injection**	1.24	0.34	1.18	0.269	0.584
	**Practical application of injections**	1.11	0.259	1.10	0.299	0.926
	**Help from medical centre and neurologists**	1.03	0.115	1.02	0.090	0.481
	**Help from caregivers**	1.08	0.272	1.05	0.123	0.710
	**Significance of treatment**	1.14	0.288	1.10	0.143	0.537

^1)^ First visit, Visit 1: start of therapy

^2)^ Last visit, Visit 5: 2 years after start of therapy

Mean was calculated based on all answers were coded as follows: 1. No problem/good support/very important; 2. Some problems/support might be better/important as a part of treatment; 3. Serious problems/no support/not too important;

* Statistically significant: p<0.05

#### B. Patient follow-up—lack of persistence

**Fig 2:**
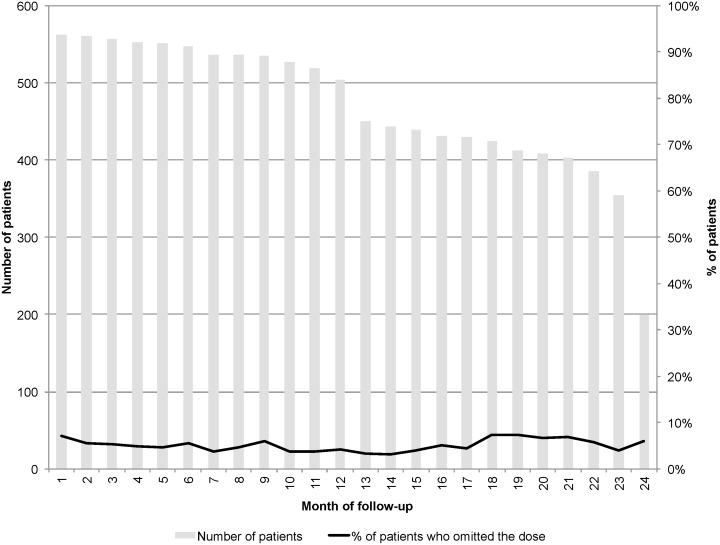
Percentage of patients who omitted at least one dose of drug reported at different time points, as captured by the RODQ.

The percentage of patients who omitted one or two doses of the drug was 0.5–5.0%, while the percentage of patients who skipped three or more doses was 0.7–5.5%. The total percentage of patients who reported omitting at least one dose of the drug at different visits was 3.2–7.2% (Fig 2).

When considering the number of omitted doses, our analysis showed that for patients who did not omit any dose or omitted a maximum of two doses (10.3%), a lower percentage stopped treatment compared with patients who omitted at least three doses (22.0%) (p = 0.016). When analysing patients who did not omit a dose compared with those who omitted at least one dose, no differences were observed: 11.5% vs 12.2%; p = 0.161.

Patients who omitted a maximum of two doses compared to those who omitted at least three doses had: lower number of relapses that occurred during the 2 years preceding starting of the study (1.99 vs. 2.38; p = 0.008), lower EDSS at the first visit (1.39 vs. 1,72; p = 0.016), the EDSS at the last visit (1.43 vs. 1.85; p = 0.009), and the mean number of visits where adverse events were reported during Betaferon therapy (0.11 vs. 0.49; p = 0.000). Moreover, patients who did not omit or who omitted a maximum of two doses received more help from their caregivers (p = 0.014).

**Fig 3:**
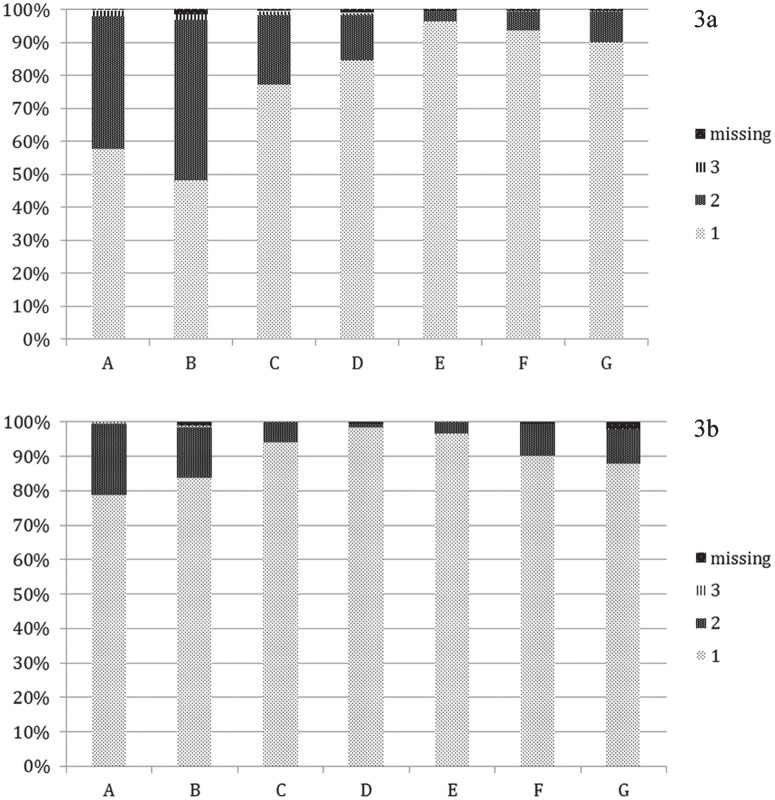
The proportion of patients with patient-reported treatment-related problems at a) The first visit; b) The last visit. Legend: A. Reported injection site reactions; B. Reported problems with other adverse events; C. Reported fear of injection; D. Reported problems with practical application of injections; E. Reported support from medical centre and neurologists; F. Reported caregivers support; G. Reported importance of the treatment with Betaferon; 1. No problem/good support/very important; 2. Some problems/support might be better/important as a part of treatment; 3. Serious problems/no support/not too important

Adverse events: the number of patients with injection site reactions differed between visits. The largest number of patients reporting injection site reactions was at Visit 1 when 231 patients from a total of 552 reported injection site reactions (41.8%). The lowest number occurred at the last visit when 43 patients from a total of 204 reported injection site reactions (21.1%) (Fig 3a and 3b). The number of patients who reported other adverse events (flu-like symptoms, fever) differed between visits. The largest number of patients reporting other adverse events was at Visit 1 (277; 50.2%), while the lowest number occurred at the last visit with only 31 patients (15.2%) (Fig 3a and 3b).

The number of patients who reported fear of injection was highest at Visit 1 with 122 patients (22.1%). The lowest number occurred at the last visit with only 12 patients (5.9%) (Fig 3a and 3b). Also, reporting of problems with the practical application of injections differed at each visit. The number of patients reporting problems injecting the treatment was highest at Visit 1: 78 patients (14.1%) (Fig 3a and 3b).

The number of patients who mentioned problems in obtaining support from a medical centre or neurologist was highest at Visit 1 with 18 patients (3.3%), and lowest at the last visit (seven patients; 3.4%) (Fig 3a and 3b). A similar tendency was seen for obtaining support from caregivers; the highest number was reported at Visit 1, with 33 patients (6.0%) expressing dissatisfaction, and the lowest number was reported at the last visit, with 19 patients (9.3%) (Fig 3a and 3b).

Almost all patients reported treatment with Betaferon as very important: 487 patients (88.2%) at Visit 1 and 179 patients (87.7%) at the last visit. Interestingly, the percentage of patients for whom treatment was very important increased at the interviews at Visits 4, 5 and 6, up to 93.6% (Fig 3a and 3b).

#### Multivariable logistic regression models analysing the risk factors associated with premature discontinuation of treatment and the risk of lack of persistence during therapy

The risk of premature discontinuation of therapy in Model 1, based on the average of experienced problems, was increased in the following groups: younger patients (r = -0.09, p = 0.012); patients with a shorter duration of the disease (r = -0.34, p = 0.028); patients with more advanced disease, as measured by the EDSS score at baseline (r = 0.85, p = 0.002); patients with more severe deterioration, as measured by the EDSS score during the study (r = 1.56, p = 0.000); patients who experienced more frequent adverse events (r = 6.97, p = 0.000) (Table 3).

**Table 3:** Multivariable logistic regression model analyzing the risk factors on premature discontinuation of treatment (Model 1).

Variable	Regression coefficient	p-value
Age	-0.09	0.012
Time since first symptoms	-0.34	0.028
EDSS First visit	0.85	0.002
EDSS difference	1.56	0.000
Number of visits with adverse events reported	6.97	0.000

The risk of omitting the dose during therapy in Model 2 was increased in the following groups of patients: those that were working or studying (r = 0.91, p = 0.008); patients with more advanced disease, as measured by the EDSS score at baseline (r = 0.46, p = 0.000); patients who experienced more frequent adverse events (r = 1.65, p = 0.004); patients who received less support from their caregivers (r = 1.36, p = 0.04) (Table 4).

**Table 4:** Multivariable logistic regression model analysing the risk of omitting the dose during therapy (Model 2).

Variable	Regression coefficient	p-value
Employment status	0.91	0.008
EDSS first visit	0.46	0.000
Number of visits with adverse events reported	1.65	0.004
Care status (from caregivers)	1.36	0.04

## Discussion

We aimed to identify the risk factors for discontinuation of IFNβ-1b (Betaferon) therapy and a lack of persistence in MS patients. We observed that the risk of premature discontinuation of therapy was increased: in younger patients; in patients with shorter duration of the disease; in patients with more advanced disease, as measured by the EDSS score at baseline; in patients with more severe deterioration, as measured by the EDSS score during the study; in patients who experienced more frequent adverse events. Patients with a greater number of relapses and progression of the disease stopped the therapy earlier.

We also analysed the factors influencing a lack of persistence. The risk of omitting the dose during therapy increased: in working or studying patients; in patients with more advanced disease, as measured by the EDSS score at baseline; in patients reporting more frequent adverse events; in patients who received less support from their caregivers.

Finally, we found no difference in clinical characteristics between patients who did not omit doses and those who omitted at least one dose. However, we did find a difference between patients who did not omit a dose or omitted a maximum of two doses and those who omitted three or more doses. In addition, we observed an increased proportion of permanent treatment discontinuation in patients who omitted three or more doses than in those who omitted fewer doses. These findings may suggest that omission of a maximum of two doses does not indicate a higher probability of treatment discontinuation, and that the patients who omit single doses are similar to those who fully adhere to treatment. Thus, it may be implied that only omission of three or more doses should be perceived as a warning sign for treatment withdrawal.

When considering age and therapy duration, the results of our study contradict the study carried out by Cunningham et al. in a Swedish population. They found that patients who were older at the beginning of treatment, and had longer disease duration, gave up therapy earlier. In their study, the patients with a high EDSS score were more likely to stop therapy [21]. Similarly, Rio et al. observed that the EDSS score at entry was the main factor that predicted discontinuation of therapy [22]. On the other hand, Tremlett et al. identified disability, gender, and disease duration as factors that influenced discontinuation of IFNβ therapy [23].

Our results are only partly in line with the study by Alroughani et al., who found that in a Kuwait population those who did not adhere to therapy had a shorter mean disease duration and a greater likelihood of having EDSS scores of 3 or lower compared with those who did adhere to therapy [24]. Inconvenience was the most common reason for non-adherence (32.9%), followed by perceived lack of efficacy (25.9%), adverse events (23.5%), and physician-documented disease progression (17.7%) [24]. Devonshire et al. reported that adherent patients had shorter disease duration, and a shorter duration of therapy, than non-adherent patients [25]. Similarly, Treadaway et al. reported a higher adherence rate for MS patients with a disease duration of less than 3 years [26].

IFNβ-1b or Betaferon is administered every other day subcutaneously by self-injection. The adverse event profile is similar to that seen with other IFNs, for example: reactions at the injection site, including injection site necrosis; flu-like symptoms; depression; elevated liver enzyme levels [27,28]. Other reactions were observed that were possibly related to IFNβ-1b therapy, such as lymphopenia, leukopenia, and granulocytopenia [29]. Non-adherence to treatment is often reported because of all of these adverse events and the chronic character of the disease. Several studies showed that 2–20% of patients on IFNβ therapy discontinued therapy during the initial 6 months [23,30–34]. Longer studies showed that 8–46% of patients stopped or switched their therapy over time [23,34–39]. In our study, 12% of patients stopped therapy within 24 months, which is placed in the lower range of the reported data. We speculate that additional nurse support may explain this beneficial finding.

Considering adverse events may be a reason for early termination of treatment, we observed that injection site reactions were reported by 20–40% of patients included in our study, and other adverse events were seen in up to half of all patients. The percentage of patients reporting adverse events in our study was much higher, especially at the beginning of treatment, than in the study by Koch-Henriksen et al. [40]. In a group of 120 patients treated with IFNβ-1b for 12 months, they reported the following adverse events at a much lower percentage: flu-like symptoms (9%), fever (5%), injection site reactions (5%), depression (4%).

In other studies, the percentage of patients with adverse events was higher than those reported by Koch-Henriksen et al. For example, in the CHAMPS study, 104 patients (54%) in the IFNβ-1b group reported flu-like syndrome [16], while in the ETOMS study, fever was reported for 43 patients (28%), myalgia for 26 patients (17%), and chills for 17 patients (11%) [41]. In the BENEFIT study, flu-like syndrome was reported for 129 patients (44%) and fever for 38 patients (13%) [40]. Later, in the follow-up phase of the BENEFIT study, it was again shown that the presence of adverse effects may influence early treatment termination. During the follow-up phase more patients in the delayed treatment group (18 patients; 12%) prematurely stopped INFβ-1b because of adverse events compared with six patients (2%) in the early treatment group [42].

Needle phobia, which affects up to 22% of the general population, may be another significant barrier to adherence to self-administered INFβ therapies among MS patients. The physical disability and cognitive impairment resulting from MS can also make self-injection and the patient’s ability to comply with treatment regimens difficult [43]. In our study, patients who stopped therapy reported a fear of injection more often than those who adhered to treatment.

Several studies on adherence to MS therapy in clinical practice have shown that patients are more likely to discontinue therapy within the first 6 months after initiation of treatment [23]. This was not observed in our population, as most patients stopped treatment in the later stages. Our study shows that constant patient support during MS treatment is important. Support offered by healthcare personnel, such as nurses, physician assistants or neurologists, may alleviate some of the problems that occur during MS therapy [24]. Support from the caregivers may also influence treatment adherence; however, we could not confirm this from the results of our study.

Important issue for the interpretation of the results is the validity of self-report about adherence. Overall, questionnaire-based studies seem to report better adherence than those based on claims data, which may be due to social desirability [39]. Self-reported adherence may be biased toward overestimation of good adherence due to recall bias or a desire to please the investigator [44]. Also Bruce et al. stated, that studies employing only self-report may underestimate poor adherence. This conclusion was based on the fact that patients reported better adherence than was indicated by medication diaries and electronic monitoring of needle disposals [45]. In our study, to minimize this risk, patients’ reports were verified by qualified nurses. Therefore we believe that our data are of adequate precision to allow for comparison between single and 2–3 events of drug omissions.

Our results should be interpreted with caution in light of the observational nature of our study and its inherent problems, such as patients lost to follow-up, missing data, and certain level of inaccuracy. Additionally, the fact that patients who remained in the study may be mostly those who benefited from treatment needs to be considered while interpreting patients’ treatment perception, e.g. the increasing proportion of patients who considered treatment as very important. However, having a large number of enrolled patients and a relatively long follow-up does compensate for the flaws of the observational data. In addition, observational studies reflect routine clinical practice much closer than typical clinical trials; therefore, we maintain that observational studies can provide valuable insight into daily patient management and contribute to its improvement. We not only report the proportion of patients who discontinued treatment or continued it irregularly but also identify patients at risk, who needed additional care in terms of education or individual support. We also report the main problems perceived by patients that may lead to problems with adherence to treatment. We observed that adverse events alone are not the only reason for discontinuation of treatment. Awareness of these other factors should influence scientifically-evaluated therapy initiation, constant care by professionals, and implementation of follow-up programmes. In conclusion, patients at a higher risk of stopping MS treatment need to be identified early to make support available to them.
